# Associations of CT-Detected Sarcopenia and Myosteatosis With Postoperative Outcomes After Emergency Abdominal Surgery: A Systematic Review and Meta-Analysis

**DOI:** 10.7759/cureus.114647

**Published:** 2026-08-17

**Authors:** Anas M Berro, Mona B Dandashli, Hesham Mohammed Haroon Taha, Mohammed Y Alayasa, Mohammad H Souaid, Ahmed Elsir Mokhtar, Asim Ahmed

**Affiliations:** 1 Faculty of Medicine, Beirut Arab University, Beirut , LBN; 2 Faculty of Medicine, Beirut Arab University, Beirut, LBN; 3 General Surgery, Red Sea University, Port Sudan, SDN; 4 Radiology, Sidra Medicine, Doha, QAT; 5 Faculty of Medicine, Beirut Arab University, West Bekaa, LBN; 6 Anatomy, Najran University, Najran, SAU; 7 Medicine and Surgery, University of Gezira, Wad Madani, SDN

**Keywords:** computed tomography, emergency abdominal surgery, emergency laparotomy, myosteatosis, sarcopenia

## Abstract

Emergency abdominal surgery, including emergency laparotomy, is associated with substantial postoperative morbidity and mortality. Preoperative computed tomography (CT), when already obtained for diagnostic purposes, can provide opportunistic information on skeletal muscle quantity and quality that may be relevant to perioperative risk assessment. This systematic review and meta-analysis evaluated the associations between CT-detected sarcopenia, myosteatosis, and related CT-derived muscle measurements and postoperative outcomes in adults undergoing emergency abdominal surgery. Eligible observational studies assessed CT-derived muscle measurements and reported at least one postoperative outcome. Ten CT studies were retained in the overall evidence synthesis. Across the available evidence, study-defined low muscle quantity (often termed sarcopenia) and low muscle quality or myosteatosis were associated with increased postoperative mortality and severe complications. Methodological and clinical heterogeneity, particularly variation in CT assessment methods and diagnostic thresholds, limited certainty. Opportunistic CT-derived muscle assessment may provide complementary risk information alongside established perioperative risk-assessment tools rather than replace them, but standardized definitions and prospective validation are required before routine clinical implementation.

## Introduction and background

Emergency abdominal surgery, including emergency laparotomy, is performed for acute intra-abdominal conditions and is associated with substantial postoperative morbidity and mortality. Unlike elective surgery, these procedures are often undertaken in patients with acute physiological derangement and with limited time for preoperative optimization. Common indications include bowel obstruction, perforation, ischemia, and peritonitis. Previous emergency laparotomy data have shown high postoperative mortality and variation in outcomes between hospitals, highlighting the need for accurate perioperative risk assessment and improved patient stratification [1,2]. In this setting, baseline skeletal muscle status may be clinically relevant as a marker of underlying vulnerability during acute operative stress.

Sarcopenia is a progressive skeletal muscle disorder in which low muscle strength is a key feature, with low muscle quantity or quality used to confirm the diagnosis in contemporary consensus frameworks [3]. Many surgical CT studies, however, use the term sarcopenia for CT-defined low muscle quantity alone. In this review, those study-specific labels were retained for reporting, while CT-derived muscle quantity and muscle quality were treated as distinct constructs. In surgical patients, low skeletal muscle quantity may reflect reduced physiological reserve, poor nutritional status, systemic illness, or other aspects of baseline vulnerability rather than acting as an isolated causal determinant of postoperative outcome. Computed tomography (CT), when already obtained as part of the diagnostic evaluation, can provide objective information on skeletal muscle quantity and quality without requiring an additional investigation. The third lumbar vertebral level, L3, is commonly used because regional muscle measurements at this level provide a practical surrogate for whole-body muscle or fat-free mass. At L3, skeletal muscle area and skeletal muscle index provide measures of muscle quantity, while muscle attenuation provides information on muscle quality [4].

The prognostic value of sarcopenia has been examined across multiple surgical specialties. A broad systematic review and meta-analysis of surgical patients found that sarcopenia was associated with poorer postoperative outcomes, including increased mortality, complications, longer hospital stay, and lower rates of discharge home [5]. However, broad surgical reviews often combine elective and emergency procedures and multiple surgical populations. Because emergency patients often present with acute illness and limited opportunity for optimization, findings from broader or predominantly elective surgical populations may not be directly applicable to emergency abdominal surgery.

Evidence specific to emergency abdominal surgery, particularly emergency laparotomy, remains limited. Broad surgical evidence supports an association between sarcopenia and adverse postoperative outcomes, but it does not fully establish whether CT-detected sarcopenia, myosteatosis, and related CT-derived muscle measurements are consistently associated with postoperative outcomes in emergency surgical populations [5]. This review addresses this specific evidence gap.

The main strength of this review is its emergency surgery-specific focus and its separation of CT-derived muscle quantity from muscle quality. By distinguishing study-defined low muscle quantity (often termed CT sarcopenia) from myosteatosis or low muscle quality, and by combining quantitative meta-analysis with narrative synthesis when pooling was not appropriate, this review provides a clinically focused synthesis of whether opportunistic CT-derived muscle assessment may provide complementary risk information for postoperative risk stratification in emergency abdominal surgery.

Therefore, the objective of this systematic review and meta-analysis was to evaluate the association between CT-detected sarcopenia, myosteatosis, and related CT-derived muscle measurements and postoperative outcomes after emergency abdominal surgery, including emergency laparotomy. The review also aimed to summarize CT measurement approaches, compare findings on muscle quantity and muscle quality, assess postoperative mortality and complication outcomes, and evaluate the methodological quality of the included observational studies.

## Review

Methods

Study Design and Reporting Framework

This systematic review and meta-analysis was conducted in accordance with the Preferred Reporting Items for Systematic Reviews and Meta-Analyses (PRISMA) 2020 statement [6]. The review question, eligibility criteria, search strategy, study selection process, data extraction approach, risk-of-bias assessment, and synthesis plan were defined before final data extraction. This review was not prospectively registered in the International Prospective Register of Systematic Reviews (PROSPERO) because screening had already commenced when registration was considered; registration at that stage would no longer have been prospective. Nevertheless, the eligibility criteria, screening process, data extraction approach, risk-of-bias assessment, and synthesis methods are reported transparently. The final literature search was conducted on June 10, 2026.

The review question was: among adults undergoing emergency abdominal surgery, including emergency laparotomy, are CT-detected sarcopenia, myosteatosis, or related CT-derived muscle measurements associated with worse postoperative outcomes compared with patients without sarcopenia, myosteatosis, or low muscle quality? The population, exposure, comparator, and outcomes (PICO) framework used to define the population, exposure, comparator, and outcomes is summarized in Table 1.

**Table 1:** PICO framework for the systematic review and meta-analysis PICO, population, exposure, comparator, and outcomes; *CT, computed tomography; ICU, intensive care unit.*

PICO component	Definition used in this review
Population	Adults aged 18 years or older undergoing emergency abdominal surgery, including emergency laparotomy, emergency midline laparotomy, or urgent exploratory laparotomy
Exposure	Study-defined CT sarcopenia or low muscle quantity, including low skeletal muscle index, low psoas muscle area or index; low psoas density; myosteatosis; low skeletal muscle radiation attenuation; skeletal muscle gauge; or related CT-derived muscle measurements
Comparator	Patients without sarcopenia, without myosteatosis, or with preserved CT-defined muscle quantity or muscle quality
Primary outcome	Postoperative mortality, with priority given to 30-day mortality when available
Secondary outcomes	Ninety-day mortality, in-hospital mortality, 1-year mortality, overall postoperative complications, severe postoperative complications, Clavien-Dindo complications, intensive care unit admission or length of stay, hospital length of stay, readmission, discharge destination, and burst abdomen

Eligibility Criteria

Eligible participants were adults aged 18 years or older undergoing emergency abdominal surgery, including emergency laparotomy, emergency midline laparotomy, or urgent exploratory laparotomy. Studies were eligible if they evaluated CT-detected sarcopenia, low skeletal muscle index, low psoas muscle area or index, low psoas density, myosteatosis, low skeletal muscle radiation attenuation, skeletal muscle gauge, or another related CT-derived muscle measurement in relation to postoperative outcomes. Where an original study used the term sarcopenia for CT-defined low muscle quantity without a muscle-strength measure, that study-specific label was retained for reporting but treated analytically as a muscle-quantity exposure. Studies meeting the core population criterion required a clearly identifiable emergency abdominal surgery population or separately reported relevant subgroup data. Mixed laparotomy cohorts not restricted to emergency surgery did not contribute to the core synthesis; when they assessed CT-derived muscle measures and a prespecified outcome of interest, they could be retained only as supplementary narrative evidence. Eligible study designs included retrospective cohort studies, prospective observational studies, registry-based studies, and case-control studies.

Studies were excluded if they evaluated elective-only surgical populations, non-abdominal surgery populations, trauma-only cohorts without separately reported emergency abdominal surgery data, frailty alone without CT-defined muscle assessment, or did not report relevant postoperative outcomes. Studies were also excluded if they were reviews, editorials, commentaries, letters, case reports, protocols without completed results, conference abstracts without completed full-text reports, duplicate reports, overlapping cohort publications without distinct outcome data, or records without sufficient relevant data for either narrative synthesis or meta-analysis. Only English-language full-text reports with sufficient relevant information were included. Methodological quality was not used as an eligibility criterion. Risk-of-bias assessment was performed after study inclusion and was used to inform interpretation of the evidence rather than to determine eligibility.

Information Sources and Search Strategy

A structured literature search was performed using PubMed/MEDLINE, Google Scholar, journal websites, DOI records, publisher pages, reference lists, and manual citation searching. Searches were conducted from database inception to June 10, 2026, with restriction to English-language human studies. The search strategy combined terms related to sarcopenia, myosteatosis, skeletal muscle index, psoas muscle index, psoas density, muscle attenuation, skeletal muscle radiation attenuation, and body composition. These were combined with terms related to emergency laparotomy, urgent exploratory laparotomy, emergency abdominal surgery, emergency general surgery, laparotomy, postoperative mortality, postoperative complications, intensive care use, hospital length of stay, discharge destination, readmission, and burst abdomen.

Google Scholar was used as a supplementary search source rather than the primary bibliographic database. Google Scholar records were screened using the default search display, and screening continued until retrieved records became clearly unrelated to the review question. Journal websites, digital object identifier (DOI) records, and publisher pages were used to verify article details, full-text availability, citation information, and DOI accuracy. Reference lists of relevant systematic reviews, meta-analyses, and eligible primary studies were manually screened to identify additional potentially eligible records. The search strategy is summarized in Table 2.

**Table 2:** Search strategy and information sources CT, computed tomography; DOI, digital object identifier. Search terms were adapted according to the search functions of each source.

Source	Search strategy or search terms	Limits or notes
PubMed/MEDLINE	(sarcopenia OR myosteatosis OR "skeletal muscle index" OR "psoas muscle index" OR "psoas density" OR "muscle attenuation" OR "skeletal muscle radiation attenuation" OR "body composition") AND ("emergency laparotomy" OR "urgent exploratory laparotomy" OR "emergency abdominal surgery" OR "emergency general surgery" OR laparotomy)	Human studies; English language; full text assessed when available
Google Scholar	sarcopenia myosteatosis emergency laparotomy CT; psoas density emergency laparotomy mortality; skeletal muscle index emergency abdominal surgery; body composition burst abdomen laparotomy	Used as a supplementary source for additional records and full-text location
Journal websites and DOI pages	Searches using the titles of potentially eligible articles identified through database searching or citation tracking	Used to verify article details, full-text access, and DOI accuracy
Reference lists and citation searching	Manual review of reference lists from relevant systematic reviews, meta-analyses, and eligible primary studies	Used to identify additional emergency laparotomy or emergency abdominal surgery studies

Study Selection Process

All retrieved records were screened in two stages. First, titles and abstracts were screened against the eligibility criteria. Second, potentially eligible full-text reports were assessed against the eligibility criteria. Duplicate records were removed using the 10Scholars platform (https://10scholarsacademy.com/). Duplicate checking was based on title, author names, publication year, DOI, journal, and publisher record.

Two reviewers independently screened titles, abstracts, and full-text reports. Disagreements about eligibility were resolved through discussion and consensus. A formal inter-reviewer agreement statistic was not calculated. When duplicate or overlapping reports appeared to describe the same cohort, the report with the most complete, peer-reviewed, and directly relevant exposure-outcome data was retained.

Data Extraction

Two reviewers independently extracted data from each included study using a predefined extraction form. Study and patient characteristics included author, year, country, setting, study design, study period, sample size, population characteristics, inclusion and exclusion criteria, surgical indication, and type of emergency operation. CT-related variables included CT timing, CT measurement level, CT software, body-composition measurement method, and the definition and cutoff used for sarcopenia or myosteatosis. Outcome-related data included the number of patients with and without sarcopenia or myosteatosis, reported outcomes, event counts, crude and adjusted effect estimates, adjustment variables, follow-up duration, and study limitations. Extracted effect estimates included odds ratios, hazard ratios, risk ratios, and 95% confidence intervals when reported. Extracted data were reviewed for completeness and consistency by both reviewers, and disagreements were resolved through discussion and consensus. Study authors were not contacted for unpublished or missing data.

Outcomes

The primary outcome was postoperative mortality, with priority given to 30-day mortality when available. Secondary mortality outcomes included in-hospital mortality, 90-day mortality, and 1-year mortality. Other secondary outcomes included overall postoperative complications, severe postoperative complications, Clavien-Dindo complications, intensive care unit admission, intensive care unit length of stay, hospital length of stay, readmission, discharge destination, and burst abdomen. Severe postoperative complications were defined according to the definitions used in each included study, most commonly Clavien-Dindo grade III or higher, Clavien-Dindo grade IIIb or higher, or equivalent major postoperative complication categories [7]. Differences in outcome definition, timing of assessment, and reporting format were considered during synthesis.

Risk-of-Bias Assessment

Because all included studies were observational, methodological quality was assessed using an adapted, domain-based approach informed by the Newcastle-Ottawa Scale [8]. For this review, the assessment considered cohort selection, applicability to the review question, ascertainment of CT-defined exposure, comparability between exposure groups, adjustment for confounding, outcome ascertainment, completeness of follow-up or outcome data, and missing CT or anthropometric data. Overall risk-of-bias judgments were expressed as lower, moderate, or high concern based on the pattern of domain-level assessments. Risk-of-bias judgments were used to guide interpretation of the evidence and were not used to exclude studies.

Data Synthesis and Statistical Analysis

All included studies were summarized narratively. Meta-analysis was performed when at least two clinically comparable studies reported extractable event data for the same exposure-outcome comparison. Study-defined CT sarcopenia or low muscle quantity was analyzed separately from myosteatosis or low muscle quality because these represent distinct CT-derived muscle constructs. Within each exposure-outcome meta-analysis, a cohort contributed only one study-specific measurement to a given construct to avoid duplicate weighting of the same participants. A cohort could contribute separately to muscle-quantity and muscle-quality analyses when distinct measurements and outcome data were available, but not more than once within the same pooled comparison. Because measurement methods and diagnostic thresholds differed across studies, pooled analyses combined study-defined binary exposure classifications at the construct level and did not assume anatomical equivalence or a common diagnostic cutoff. Alternative measurements were summarized narratively when they were not selected for pooled comparison.

For dichotomous outcomes, crude odds ratios with 95% confidence intervals were calculated from available event counts [9]. Random-effects models were used because clinical and methodological heterogeneity was expected among the included studies [10]. Heterogeneity was assessed using the I² statistic [11]. Adjusted effect estimates were summarized separately from crude event-based meta-analysis because adjustment variables differed between studies. Publication bias was not assessed using funnel plots because fewer than 10 studies were included in each pooled analysis [12].

Subgroup and Sensitivity Considerations

Where appropriate, studies restricted to emergency laparotomy formed the core evidence base. Broader emergency abdominal surgery studies could contribute to quantitative synthesis when their populations, exposures, outcomes, and extractable event data were clinically comparable with the relevant analysis. Mixed laparotomy cohorts not restricted to emergency surgery were used only for supplementary narrative interpretation when clinically relevant to the review question. Potential sources of heterogeneity considered during interpretation included emergency laparotomy-only versus broader emergency abdominal surgery populations, psoas-derived versus total L3 skeletal muscle measurements, muscle quantity versus muscle quality, short-term versus longer-term mortality, and adjusted versus unadjusted estimates. No formal subgroup or sensitivity meta-analyses were undertaken because the number of studies within candidate comparisons was insufficient for reliable analysis; these factors were considered qualitatively during interpretation. Because the review included observational studies, causal conclusions were avoided.

Certainty of Evidence and Ethics

A formal GRADE assessment was not performed. The certainty of evidence was interpreted narratively based on study design, consistency of findings, risk of bias, sample size, directness, clinical applicability, and heterogeneity in CT measurement methods and outcome definitions. Ethics approval was not required because this systematic review used previously published aggregate data and did not involve collection of individual patient-level data.

Results

Study Selection

The database and supplementary searches identified 318 records. After removal of 94 duplicate records, 224 records were screened by title and abstract. Of these, 183 records were excluded because they were not relevant to the review question, did not include emergency laparotomy or emergency abdominal surgery populations, did not assess CT-defined sarcopenia, myosteatosis, or related CT-derived muscle measurements, or were not eligible primary research articles. Full-text reports of 41 articles were sought for retrieval. Four reports could not be retrieved, and 37 full-text reports were assessed for eligibility. Following full-text assessment, 27 reports were excluded because of the wrong population, exposure, or outcome (n = 14), the wrong publication type or study design (n = 11), or duplicate or overlapping cohort data (n = 2).

**Figure 1:**
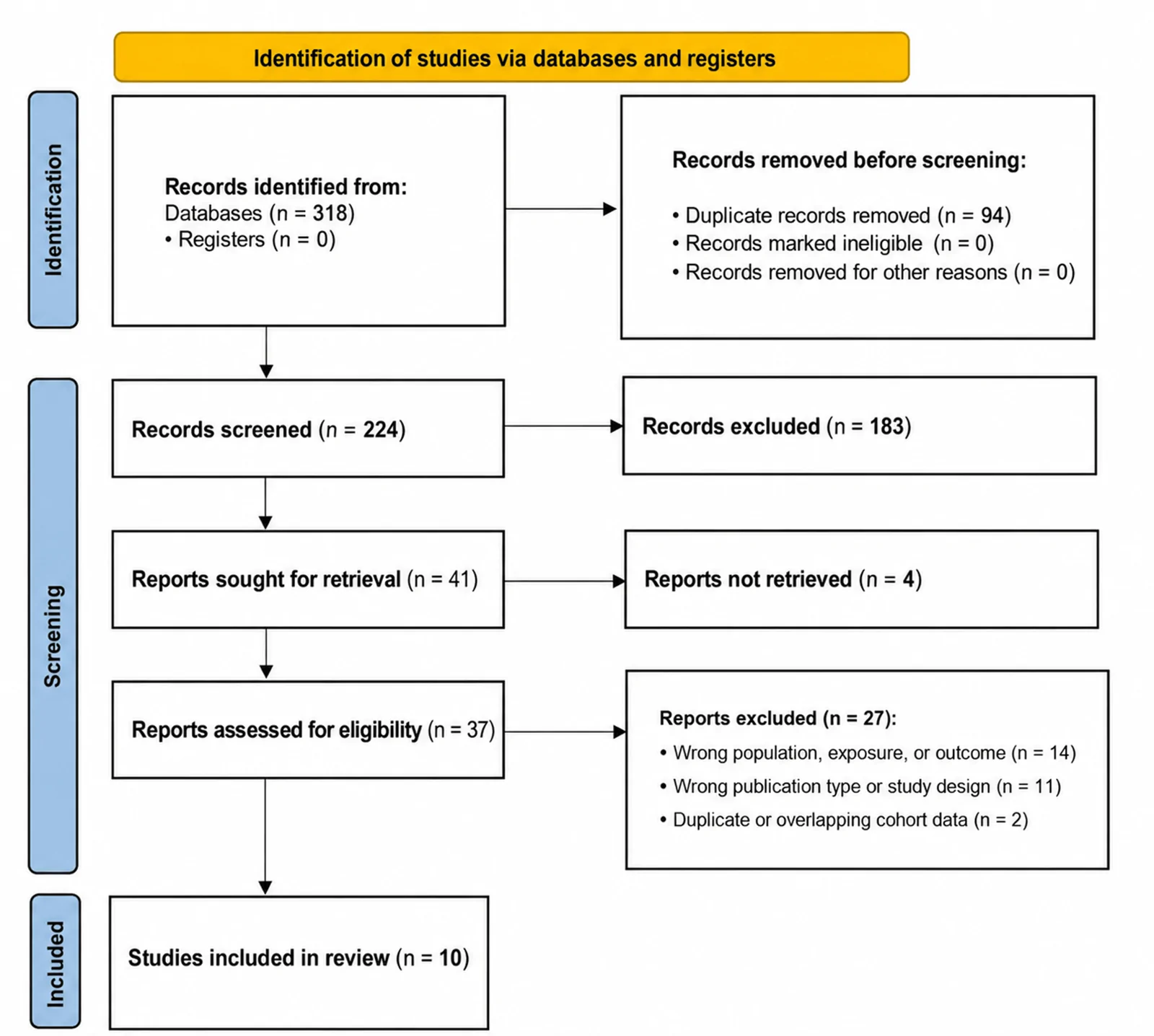
PRISMA flow diagram of study selection PRISMA, Preferred Reporting Items for Systematic Reviews and Meta-Analyses. The figure summarizes record identification, duplicate removal, title and abstract screening, full-text assessment, exclusion reasons, and final inclusion in the review.

Ten CT-based studies were retained in the overall evidence synthesis [13-22]. Nine studies met the core emergency abdominal surgery population criterion and evaluated CT-defined sarcopenia, myosteatosis, or related CT-derived muscle measurements [13-19,21,22]. Emergency laparotomy-specific studies formed the core evidence, while Kubo et al. [22] contributed broader emergency abdominal surgery evidence. Meyer et al. [20] was retained for supplementary narrative synthesis only because it assessed CT-defined body composition and burst abdomen after laparotomy in a cohort that was not restricted to emergency surgery; it did not contribute to the main pooled analyses. The study selection process is summarized in Figure 1.

Study Characteristics 

The 10 CT studies described in the overall evidence synthesis were published between 2018 and 2026 and included 4,183 patients. Sample sizes ranged from 103 to 1,090 patients. Most studies were retrospective observational cohorts, although several used prospectively maintained audit or registry databases. The studies were conducted in the United Kingdom (including Scotland and Wales), Australia, China, Italy, Germany, and Japan. Nine studies met the core emergency abdominal surgery population criterion [13-19,21,22], most of which directly evaluated emergency laparotomy. Kubo N et al. [22] evaluated emergency surgery for perforation panperitonitis and contributed as broader emergency abdominal surgery evidence rather than emergency-laparotomy-specific evidence. Meyer HJ et al. [20] was included only in the supplementary narrative synthesis of burst abdomen because the mixed laparotomy cohort was not restricted to emergency surgery. The main characteristics, CT assessment methods, and key extractable outcome data are summarized in Table 3.

**Table 3:** Characteristics, CT body-composition assessment, and key outcome data of included studies CT, computed tomography; L3, third lumbar vertebra; OR, odds ratio; PBSA, psoas body surface area; PMD, psoas muscle density; PMI, psoas muscle index; PML3, psoas muscle-to-L3 vertebral body ratio; SAT, subcutaneous adipose tissue; SMI, skeletal muscle index; SMRA, skeletal muscle radiation attenuation; TPG, total psoas gauge; VAT, visceral adipose tissue; VATI, visceral adipose tissue index. Exposure-group denominators may be smaller than the total cohort size because the availability of analyzable CT images, anthropometric data, and specific body-composition measurements varied by study.

Citation	Country	Population and sample size	CT body-composition assessment	Exposure prevalence or group size	Key extractable outcomes	Role in synthesis
Trotter et al. [13]	United Kingdom	Emergency laparotomy; n = 259 for psoas density and n = 248 for psoas area	Psoas density and psoas area at L3	Low psoas density: 64/259; low psoas area: 61/248	Low psoas area: 30-day mortality 13/61 vs 17/187; 1-year mortality 26/61 vs 39/187. Low psoas density: 30-day mortality 19/64 vs 17/195; 1-year mortality 37/64 vs 36/195.	Quantitative mortality synthesis
Ming et al. [14]	Australia	Emergency laparotomy across four hospitals; n = 500	Psoas muscle-to-L3 vertebral body ratio	Dichotomous event data not fully extractable	Overall mortality: 30-day, 10.0%; 90-day, 13.4%; 365-day, 22.4%	Narrative synthesis
Wu et al. [15]	China	Adult emergency laparotomy; n = 228	PMI, PML3, PMD, TPG, and PBSA at L3	Sarcopenia by PML3: 56/228	Thirty-day mortality: 19/56 vs 13/172; Clavien-Dindo grade ≥2 complications: 33/56 vs 56/172; Clavien-Dindo grade ≥3 complications: 13/56 vs 21/172	Quantitative mortality and complications synthesis
Linganathan et al. [16]	United Kingdom (Wales)	Emergency laparotomy for non-traumatic abdominal pathology; n = 818	Psoas muscle index at L3	Sarcopenia: 83/818	Overall 30-day mortality: 57/818; in-hospital or 90-day mortality: 77/818	Narrative and prediction model synthesis
Giudici et al. [17]	Italy	Emergency laparotomy for abdominal emergencies; n = 242	SMI and muscle radiation attenuation at L3	Sarcopenia: 103/242; myosteatosis: 189/242	Sarcopenia and 30-day mortality: 27/103 vs 29/139; myosteatosis and 30-day mortality: 48/189 vs 8/53; severe complications by myosteatosis: 70/189 vs 12/53	Quantitative and narrative synthesis
Evans et al. [18]	United Kingdom	Emergency non-trauma laparotomy; n = 1,090	SMI, SMRA, and skeletal muscle gauge at L3	Low skeletal muscle gauge: 412/1,090	Overall 30-day mortality: 109/1,090; adjusted OR for low skeletal muscle gauge and 30-day mortality: 2.12; adjusted OR for 90-day mortality: 2.64	Adjusted mortality and narrative synthesis
Thu et al. [19]	United Kingdom (Scotland)	Emergency laparotomy; n = 215	Total psoas index at L3	Sarcopenia: 54/215	Thirty-day mortality: 2/54 vs 6/161; 90-day mortality: 3/54 vs 13/161; 1-year mortality: 6/54 vs 18/161	Quantitative mortality synthesis
Meyer et al. [20]	Germany	Mixed laparotomy cohort from wound infection registry; n = 118	SMI, VAT, SAT, and VATI at L3	Burst abdomen: 92/118; no burst abdomen: 26/118	Sarcopenia by Prado cutoff: 46/92 vs 5/26; adjusted OR for burst abdomen: 5.94	Supplementary narrative synthesis only
Body et al. [21]	United Kingdom	Multicentre emergency laparotomy across 10 hospitals; n = 610	SMI and skeletal muscle radiation attenuation at L3	Sarcopenia: 179; no sarcopenia: 357; myosteatosis: 195; no myosteatosis: 386	Sarcopenia and 30-day mortality: 17/179 vs 13/357; sarcopenia and 1-year mortality: 49/179 vs 41/357; myosteatosis and 30-day mortality: 29/195 vs 13/386	Core quantitative and narrative synthesis
Kubo et al. [22]	Japan	Emergency surgery for perforation panperitonitis; n = 103	SMI at L3	Sarcopenia: 50/103	Overall complications: 34/50 vs 24/53; severe complications: 14/50 vs 5/53; in-hospital mortality: 10/50 vs 3/53	Broader emergency abdominal surgery evidence; quantitative complications synthesis

Key Findings and Meta-analysis

CT-derived muscle assessment was most commonly performed at the L3 level. Low muscle quantity was defined using study-specific CT measurements, including total L3 skeletal muscle index and psoas-derived measures such as psoas muscle index, total psoas index, psoas area, or psoas-to-L3 vertebral body ratio. These measurements were not treated as anatomically equivalent. For meta-analysis, study-defined binary classifications of low muscle quantity were pooled only when the study populations, outcomes, and available event data were clinically comparable; the pooled estimand therefore reflected the association of study-defined low muscle quantity with outcome rather than a common anatomical measurement or diagnostic threshold. Low muscle quality was assessed using attenuation-based measures such as skeletal muscle radiation attenuation or psoas density. Skeletal muscle gauge, which integrates muscle quantity and attenuation, was treated as a composite measure and summarized separately rather than pooled as a pure muscle-quality exposure. Muscle-quantity and muscle-quality analyses were kept separate. Meta-analysis was performed for outcomes with at least two clinically comparable studies and extractable event counts. The pooled results are summarized in Table 4.

**Table 4:** Summary of meta-analysis results CI, confidence interval; I², inconsistency statistic; OR, odds ratio. Random-effects models were used for all pooled analyses. Study-defined CT sarcopenia or low muscle quantity analyses were kept separate from myosteatosis or low muscle quality analyses.

Outcome	Included studies	Number of studies	Pooled OR	95% CI	I²	Figure panel
Study-defined CT sarcopenia or low muscle quantity and 30-day mortality	Trotter et al. [13]; Wu et al. [15]; Giudici et al. [17]; Thu et al. [19]; Body et al. [21]	5	2.48	1.36–4.52	62.2%	Figure 2A
Study-defined CT sarcopenia or low muscle quantity and 1-year mortality	Trotter et al. [13]; Thu et al. [19]; Body et al. [21]	3	2.31	1.36–3.92	49.5%	Figure 2B
Myosteatosis or low muscle quality and 30-day mortality	Trotter et al. [13]; Giudici et al. [17]; Body et al. [21]	3	3.62	2.06–6.36	42.4%	Figure 3A
Myosteatosis or low muscle quality and 1-year mortality	Trotter et al. [13]; Body et al. [21]	2	4.10	2.06–8.18	70.8%	Figure 3B
Study-defined CT sarcopenia or low muscle quantity and severe postoperative complications	Wu et al. [15]; Giudici et al. [17]; Body et al. [21]; Kubo et al. [22]	4	1.72	1.27–2.33	0.0%	Figure 4A
Myosteatosis and severe postoperative complications	Giudici et al. [17]; Body et al. [21]	2	1.79	1.23–2.60	0.0%	Figure 4B

Mortality Outcomes

**Figure 2:**
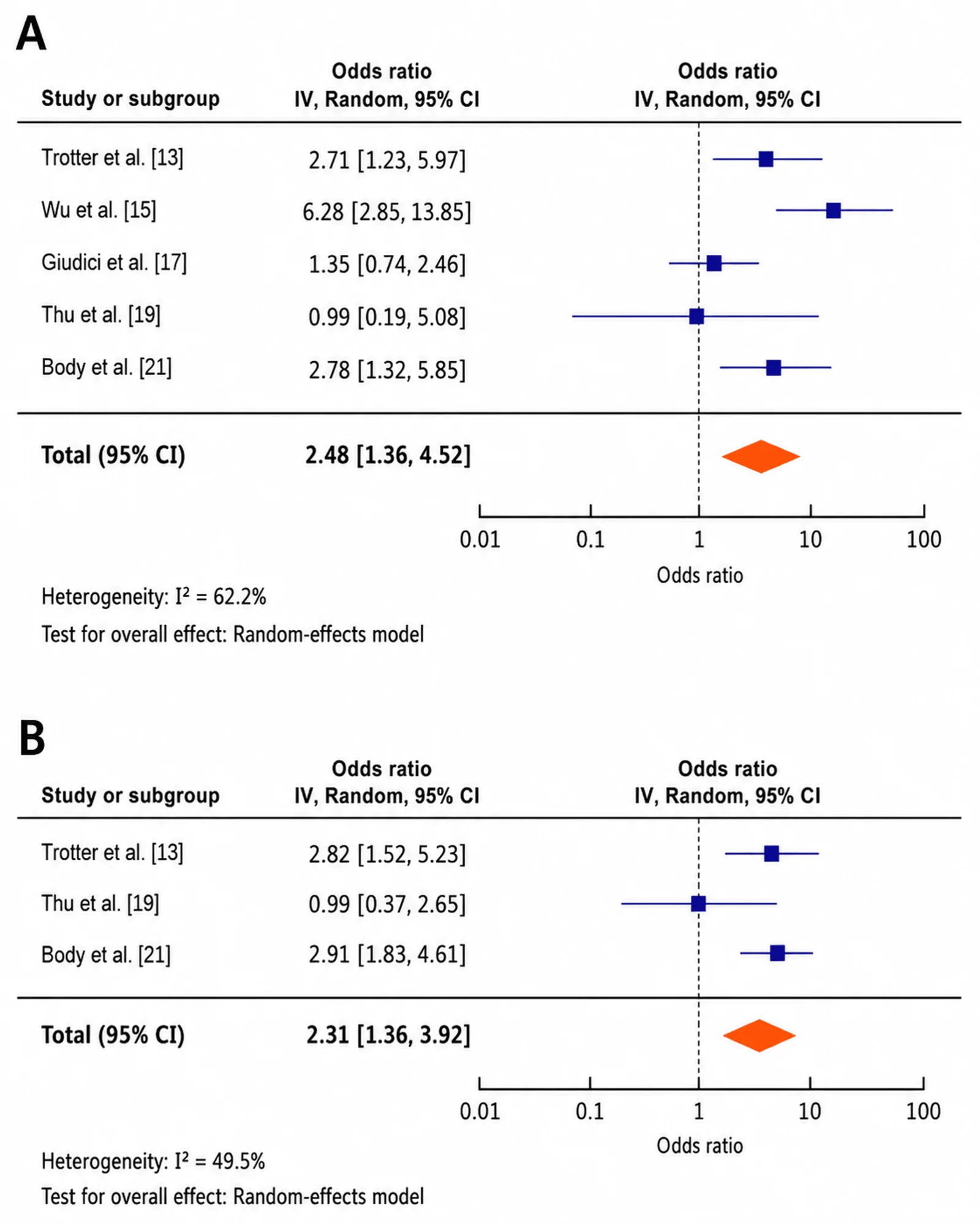
Forest plots for sarcopenia or low muscle mass and mortality outcomes Random-effects meta-analyses of mortality outcomes according to study-defined CT sarcopenia or low muscle quantity. Panel A shows 30-day mortality. Panel B shows 1-year mortality. CI, confidence interval; CT, computed tomography; OR, odds ratio.

Five studies contributed data to the meta-analysis of study-defined CT sarcopenia or low muscle quantity and 30-day mortality [13,15,17,19,21]. This exposure was associated with increased 30-day mortality, with a pooled odds ratio (OR) of 2.48 (95% CI, 1.36-4.52; I² = 62.2%), as shown in Figure 2A. Three studies reported 1-year mortality for this exposure [13,19,21], with a pooled OR of 2.31 (95% CI, 1.36-3.92; I² = 49.5%), as shown in Figure 2B.

**Figure 3:**
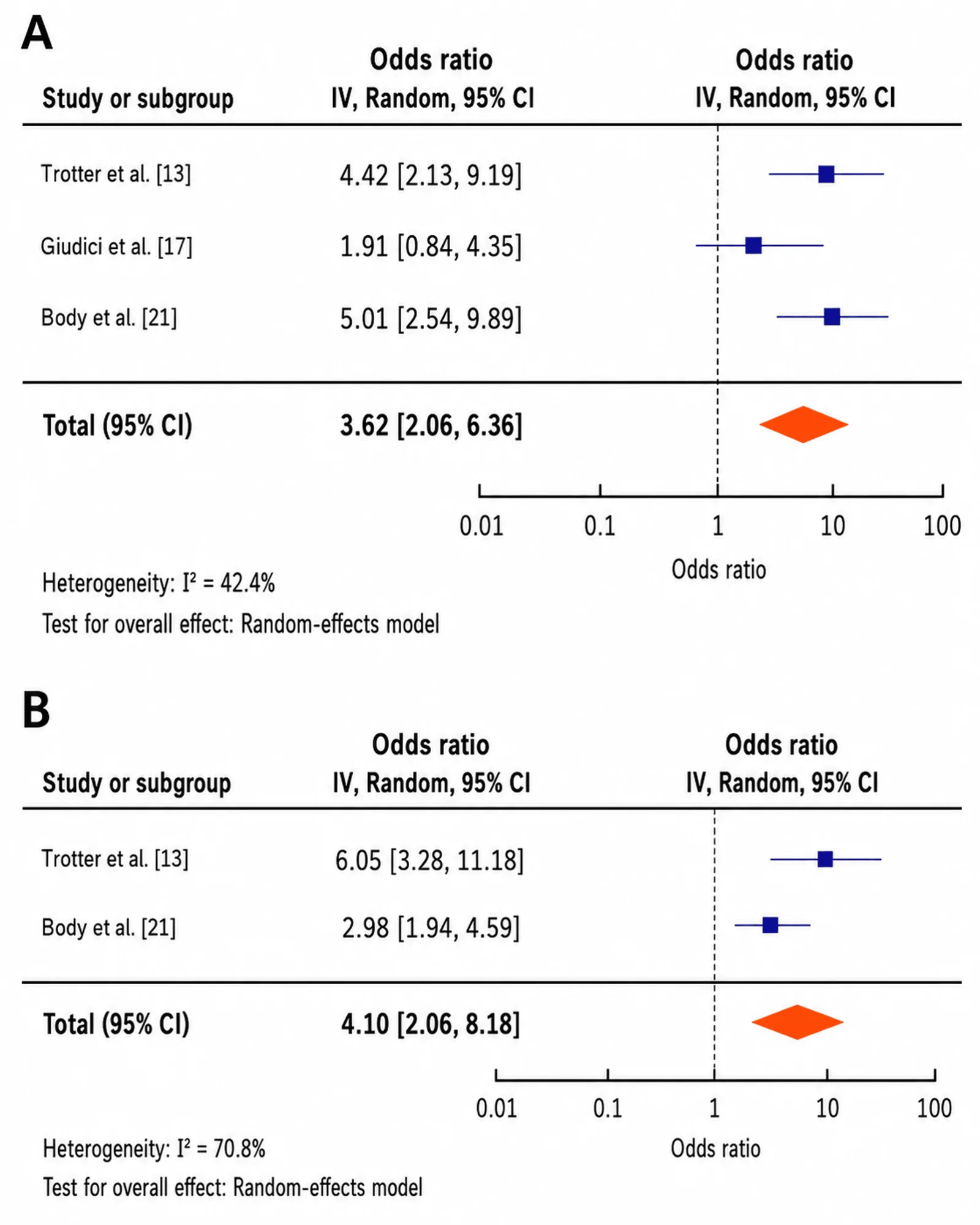
Forest plots for myosteatosis or low muscle quality and mortality outcomes Random-effects meta-analyses of mortality outcomes stratified by CT-defined myosteatosis or low muscle quality. Panel A shows 30-day mortality. Panel B shows 1-year mortality. CI, confidence interval; CT, computed tomography; OR, odds ratio.

Three studies contributed data to the meta-analysis of myosteatosis or low muscle quality and 30-day mortality [13,17,21]. Myosteatosis or low muscle quality was associated with increased 30-day mortality, with a pooled OR of 3.62 (95% CI, 2.06-6.36; I² = 42.4%), as shown in Figure 3A. Two studies reported 1-year mortality according to myosteatosis or low muscle quality status [13,21]. Myosteatosis or low muscle quality was associated with increased 1-year mortality, with a pooled OR of 4.10 (95% CI, 2.06-8.18; I² = 70.8%), as shown in Figure 3B.

Severe Postoperative Complications 

**Figure 4:**
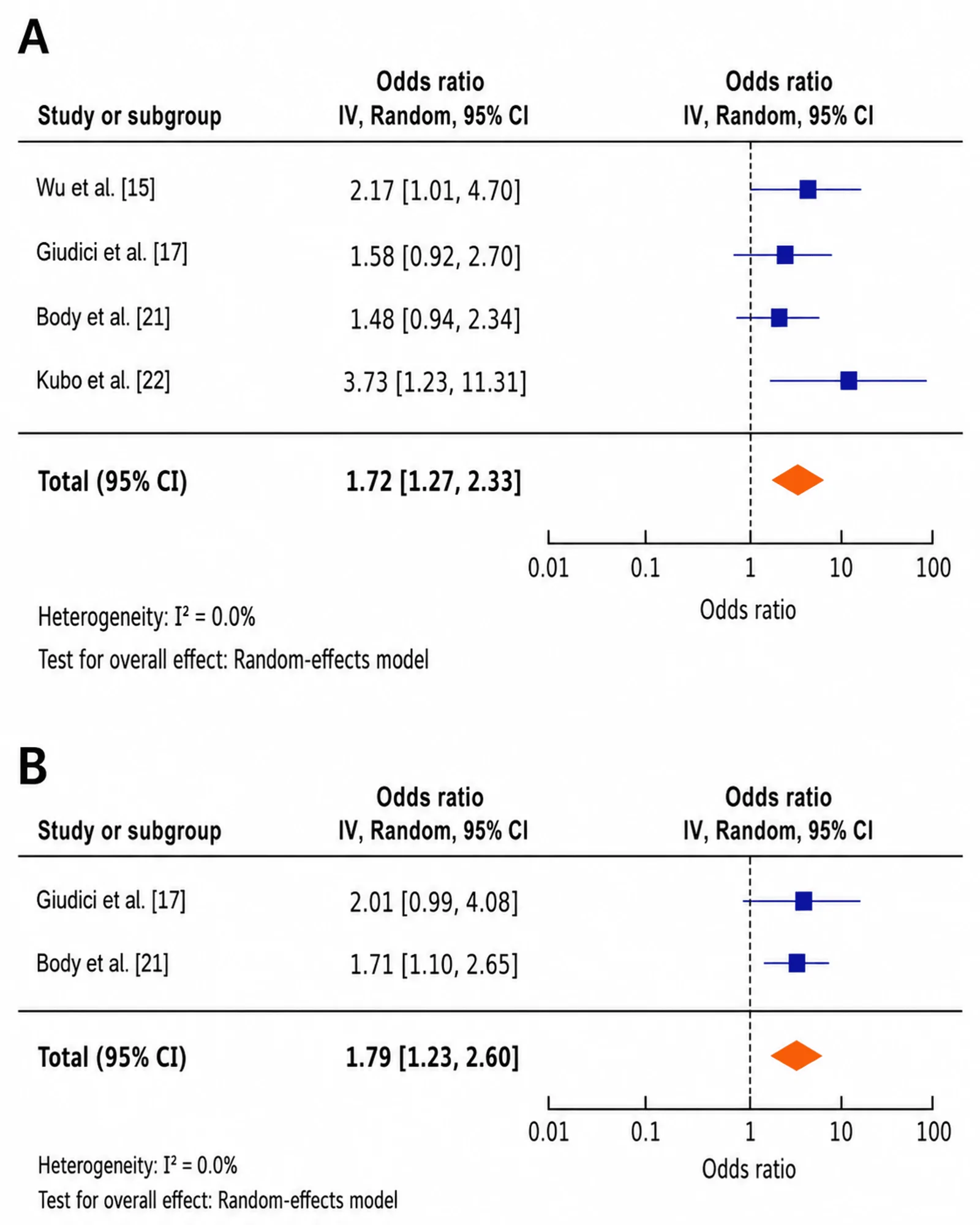
Forest plots for severe postoperative complications Random-effects meta-analyses of severe postoperative complications for study-defined CT sarcopenia or low muscle quantity and for myosteatosis. Panel A shows the association between study-defined CT sarcopenia or low muscle quantity and severe postoperative complications. Panel B shows the association between myosteatosis and severe postoperative complications. Squares represent individual study odds ratios, horizontal lines represent 95% confidence intervals, and diamonds represent pooled odds ratios. CI, confidence interval; CT, computed tomography; OR, odds ratio.

Four studies reported extractable severe postoperative complication data for study-defined CT sarcopenia or low muscle quantity [15,17,21,22]. Study-defined CT sarcopenia or low muscle quantity was associated with increased severe postoperative complications, with a pooled OR of 1.72 (95% CI, 1.27-2.33; I² = 0.0%), as shown in Figure 4A. Two studies reported severe postoperative complication data for myosteatosis [17,21]. For myosteatosis, the pooled OR was 1.79 (95% CI, 1.23-2.60; I² = 0.0%), as shown in Figure 4B.

Narrative and Supplementary Findings

Several clinically relevant findings could not be pooled because of differences in exposure definitions, outcome reporting, or availability of extractable event counts. Evans et al. [18] reported adjusted associations between low skeletal muscle gauge and 30-day and 90-day mortality. Accordingly, this study contributed to the adjusted and narrative synthesis. Ming et al. [14] reported an association between lower psoas muscle-to-L3 vertebral body ratio and mortality, but exposure-specific event counts were not fully extractable for the main pooled comparisons. Linganathan et al. [16] evaluated sarcopenia as part of the HAS prediction model, but independent sarcopenia event data were not available for pooling.

Length of stay, intensive care unit admission, discharge destination, and readmission were reported inconsistently across the included studies. Giudici et al. [17] and Body et al. [21] reported findings suggesting an association between myosteatosis and longer hospital stay, and Body S et al. [21] also reported poorer discharge outcomes among patients with myosteatosis. Meyer et al. [20] reported an association between CT-defined sarcopenia, visceral obesity, and postoperative burst abdomen. This evidence was considered supplementary because the cohort was selected from a mixed laparotomy wound infection registry rather than a population restricted to emergency surgery.

Risk of Bias

The methodological quality of the included observational studies was assessed using an adapted, domain-based approach informed by the Newcastle-Ottawa Scale [8]. For this review, the assessment considered cohort selection, applicability to the review question, ascertainment of CT-defined exposure, comparability between exposure groups, adjustment for confounding, outcome ascertainment, completeness of follow-up or outcome data, and missing CT or anthropometric data. Missing CT, anthropometric, or follow-up data were considered within the missing-data/follow-up domain and contributed to study-level judgments when present. Risk-of-bias judgments were used to guide interpretation of the evidence and were not used as exclusion criteria.

Overall, most included studies were judged to have moderate concern. Evans RPT et al. [18] and Body S et al. [21] had the lowest overall risk-of-bias judgments among the included studies. Several studies had moderate concerns because of retrospective design, single-centre or single-trust recruitment, heterogeneous emergency surgical indications, study-specific CT measurement approaches, non-standardized sarcopenia or myosteatosis cutoffs, exclusions because of missing or inadequate CT imaging, missing anthropometric or body-composition data, and incomplete adjustment for confounding. These limitations were particularly relevant to Trotter et al. [13], Wu et al. [15], Giudici et al. [17], Thu et al. [19], and Kubo et al. [22].

Multicentre or larger emergency laparotomy cohorts generally had lower concern for selection and outcome ascertainment, but some still had moderate concern for exposure assessment or confounding because CT thresholds, measurement methods, or model adjustment variables differed across studies. Ming et al. [14] and Linganathan et al. [16] had relatively strong cohort-level features, but their contribution to pooled estimates was limited by non-standardized exposure definitions, prediction-model focus, or lack of fully extractable exposure-specific event data.

**Figure 5:**
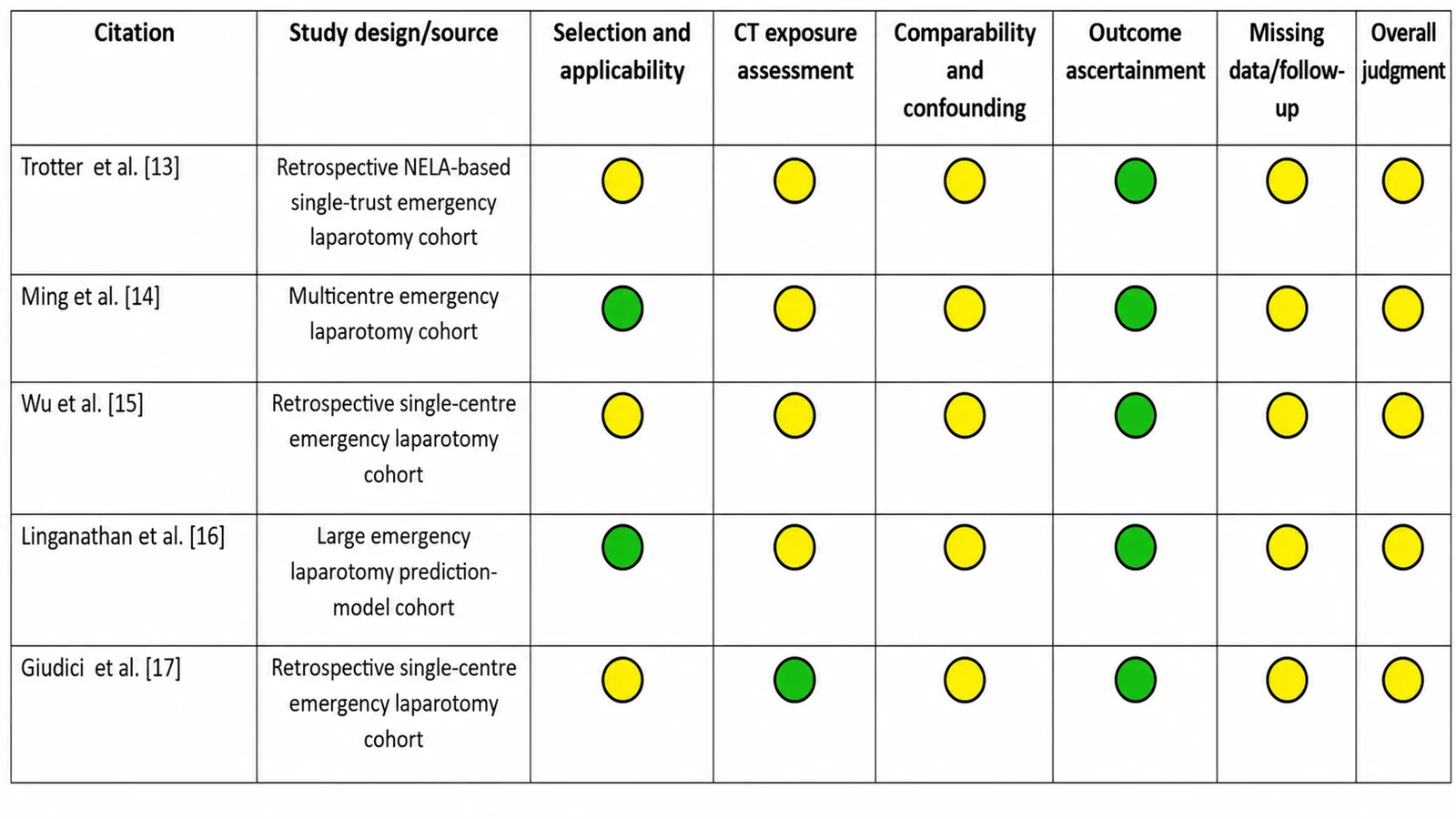
Traffic-light risk-of-bias assessment of included observational studies (Part 1) Traffic-light assessment of risk of bias for included observational studies [13-17]. 🟢 indicates low concern, 🟡 indicates moderate concern, and 🔴 indicates high concern. For the overall judgment column, 🟢🟡 indicates lower concern, 🟡 indicates moderate concern, and 🔴 indicates great concern for the primary review question. Domains included selection and applicability, CT exposure assessment, comparability and confounding, outcome ascertainment, and missing data or follow-up completeness. CT, computed tomography; NELA, National Emergency Laparotomy Audit.

Meyer et al. [20] was judged to have great concern for selection and applicability to the primary review question because the cohort was derived from a mixed laparotomy wound infection registry and was not restricted to emergency surgery. Although the study used CT-defined body-composition measures and reported multivariable modelling for burst abdomen, its applicability to the main emergency abdominal surgery mortality and complication outcomes was limited. Therefore, this study was retained only for supplementary narrative synthesis. The study-level traffic-light risk-of-bias assessments are shown in the figures given below. Figure 5 summarizes studies [13-17].

**Figure 6:**
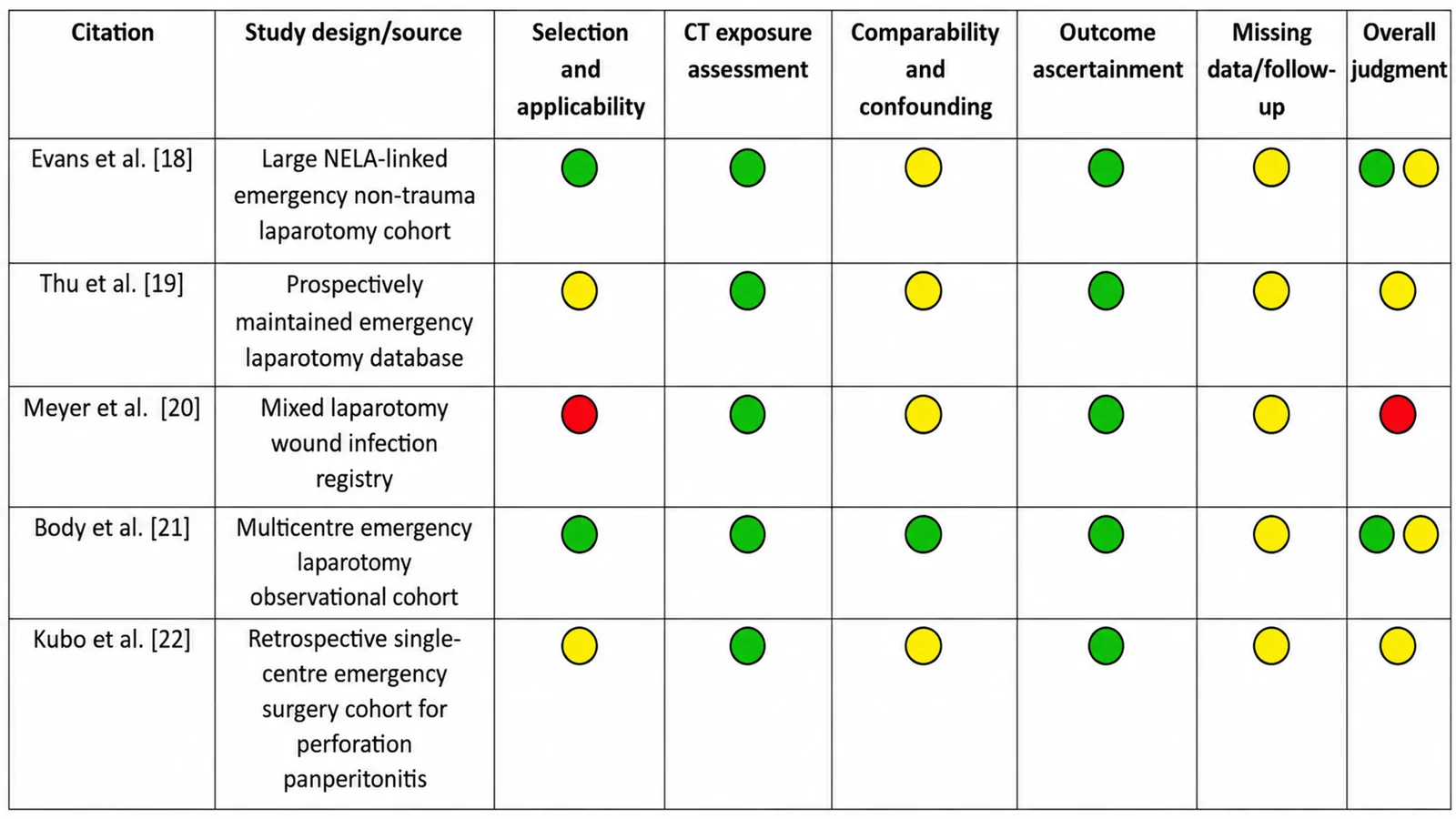
Traffic-light risk-of-bias assessment of included observational studies (Part 2) Traffic-light assessment of risk of bias for included observational studies [18-22]. 🟢 indicates low concern, 🟡 indicates moderate concern, and 🔴 indicates high concern. For the overall judgment column, 🟢🟡 indicates lower concern, 🟡 indicates moderate concern, and 🔴 indicates high concern for the primary review question. Meyer et al. [20] was judged to have high concern for selection and applicability because the cohort was not restricted to emergency surgery. Domains included selection and applicability, CT exposure assessment, comparability and confounding, outcome ascertainment, and missing data or follow-up completeness. CT, computed tomography; NELA, National Emergency Laparotomy Audit.

The remaining study-level traffic-light risk-of-bias assessments are shown in Figure 6.

Across methodological domains, outcome ascertainment was the strongest domain because mortality and major postoperative outcomes were generally objectively defined or obtained from clinical records, audit databases, registries, or follow-up systems. In contrast, comparability and confounding were the most frequent source of moderate concern because most studies were observational and differed in the variables included in adjusted models. The missing-data/follow-up domain was rated as moderate concern in all 10 studies, reflecting exclusions related to unavailable or unsuitable CT imaging, missing anthropometric data, incomplete body-composition measurements, or incomplete follow-up/reporting. A summary of domain-level judgments is provided in Table 5.

**Table 5:** Summary of risk-of-bias judgments across methodological domains 🟢 low concern; 🟡 moderate concern; 🔴 high concern. Percentages were calculated using 10 included studies as the denominator.

Domain	🟢 n (%)	🟡 n (%)	🔴 n (%)
Selection and applicability	4 (40%)	5 (50%)	1 (10%)
CT exposure assessment	6 (60%)	4 (40%)	0 (0%)
Comparability and confounding	1 (10%)	9 (90%)	0 (0%)
Outcome ascertainment	10 (100%)	0 (0%)	0 (0%)
Missing data/follow-up	0 (0%)	10 (100%)	0 (0%)

Discussion

Principal Findings

This systematic review and meta-analysis found that study-defined low muscle quantity (often termed CT sarcopenia), myosteatosis, and related CT-derived muscle abnormalities were associated with worse postoperative outcomes after emergency abdominal surgery, including emergency laparotomy. The clearest findings involved mortality and severe postoperative complications. Study-defined low muscle quantity (often termed CT sarcopenia) was associated with increased 30-day and 1-year mortality, while myosteatosis or low muscle quality showed similar adverse associations with mortality despite being evaluated in fewer studies [13,15,17,19,21]. Severe postoperative complications were also more frequent among patients with low muscle quantity or myosteatosis [15,17,21,22].

The findings were not uniform across all studies. Thu et al. [19] reported weaker or non-significant associations between total psoas index-defined sarcopenia and mortality, whereas Trotter et al. [13], Wu et al. [15], and Body et al. [21] reported stronger associations between reduced muscle quantity or quality and mortality. This variation may reflect differences in patient populations, CT measurement methods, diagnostic thresholds, baseline risk, surgical indications, and outcome definitions.

Interpretation of Findings

The association between low muscle quantity and adverse postoperative outcomes is clinically plausible. Emergency laparotomy represents substantial physiological stress and is commonly performed for acute abdominal conditions including obstruction, perforation, ischemia, sepsis, and peritonitis. Reduced skeletal muscle quantity may reflect lower baseline muscle mass and poorer nutritional status. Acute inflammation and catabolic stress may further increase vulnerability during postoperative recovery. These factors may partly explain the higher mortality and complication rates reported among patients with study-defined CT sarcopenia or low muscle quantity [13,15,17,21,22]. Because the included studies were observational, these findings should be interpreted as associations rather than evidence of causation.

Muscle quality represents a different CT-derived characteristic from muscle quantity. Myosteatosis, reduced skeletal muscle attenuation, and reduced psoas density reflect aspects of muscle composition and quality rather than muscle size alone. Skeletal Muscle Gauge is a composite CT-derived measure that incorporates both skeletal muscle quantity and muscle attenuation. Studies evaluating myosteatosis or other low-muscle-quality measures reported adverse associations with mortality and severe postoperative complications [13,17,21]. Body et al. [21] reported an association between myosteatosis and 30-day and 1-year mortality after adjustment for National Emergency Laparotomy Audit (NELA)-predicted mortality, while Giudici et al. [17] reported associations with severe complications and longer hospital stay. These findings support considering muscle quantity and muscle quality as related but distinct CT-derived characteristics.

The included evidence was insufficient to draw firm conclusions regarding prolonged ICU stay, ventilator-weaning outcomes, or delayed functional recovery because these outcomes were not reported consistently enough for synthesis.

Comparison With Included Evidence

The included studies used different CT-based definitions and measurement approaches. Some evaluated localized psoas-derived measurements, including psoas area, psoas density, psoas muscle index, total psoas index, or psoas muscle-to-L3 vertebral body ratio [13-16,19]. Other studies evaluated total skeletal muscle cross-sectional area at L3 using skeletal muscle index, skeletal muscle radiation attenuation, or composite measures incorporating muscle quantity and attenuation [17,18,21,22].

These measurements should not be considered anatomically interchangeable. A localized psoas cross-sectional measurement differs from assessment of the total skeletal muscle cross-sectional area at L3. Similarly, attenuation-based measurements primarily characterize muscle quality, whereas skeletal muscle index and most psoas-derived indices characterize muscle quantity. Skeletal Muscle Gauge combines information on muscle quantity and attenuation and therefore represents a composite measure. Terminology for muscle quantity and muscle quality was kept distinct throughout this review.

The larger cohorts and those with lower overall risk-of-bias judgments generally supported the clinical relevance of CT-derived muscle assessment. Body et al. [21] evaluated a multicentre emergency laparotomy cohort and reported associations between sarcopenia, myosteatosis, morbidity, 30-day mortality, and 1-year mortality. Evans et al. [18] reported that low skeletal muscle gauge was independently associated with 30-day and 90-day mortality in a large emergency laparotomy cohort. Wu et al. [15] found that a psoas-based CT measure remained associated with 30-day mortality after adjustment. In contrast, Ming et al. [14] and Linganathan et al. [16] were summarized narratively because their data structures did not allow direct inclusion in the main event-count meta-analyses.

Kvist et al. [23] was considered only as contextual background regarding burst abdomen. Because this study did not assess CT-defined sarcopenia, myosteatosis, or other CT-derived muscle measurements, it did not contribute to the included CT evidence or pooled analyses.

Clinical Implications

Preoperative CT imaging that has already been obtained for diagnostic purposes may provide complementary information relevant to perioperative risk assessment in emergency surgical patients. Emergency laparotomy frequently requires rapid decision-making, and CT-derived muscle measurements may be considered alongside established approaches such as NELA risk estimates, American Society of Anesthesiologists (ASA) grade, frailty assessment, and comorbidity-based evaluation [16,18,21].

These findings do not support using CT-derived low muscle quantity or myosteatosis as standalone criteria for denying surgical intervention. Instead, CT-derived muscle information may support shared decision-making and perioperative planning when interpreted together with clinical status and established risk-assessment tools. It may also inform discussions about postoperative monitoring and supportive-care planning, but the clinical benefit of management pathways triggered by CT-derived muscle findings was not established by the included evidence. Prospective studies are required to determine whether such targeted pathways improve clinical outcomes.

Methodological Considerations

Heterogeneity was the main methodological challenge. The included studies differed in CT measurement level, muscle group assessed, software, cutoff values, exposure definitions, and outcome reporting. Some studies used sex-specific quartiles or tertiles, whereas others applied previously published thresholds [13,15,17,19,21,22]. Methods used to characterize muscle quality also differed. Trotter et al. [13] used psoas density, Body et al. [21] and Giudici et al. [17] evaluated skeletal muscle attenuation, and Evans et al. [18] used Skeletal Muscle Gauge, a composite measure incorporating muscle quantity and attenuation.

Muscle quantity and muscle quality were therefore synthesized separately. This reduced the risk of combining methodologically and biologically different exposures and improved interpretability. Within the muscle-quantity analyses, study-specific classifications were retained rather than assuming anatomical equivalence between psoas-derived and total L3 skeletal muscle measurements.

Differences in data structure also affected quantitative synthesis. Ming et al. [14] reported relevant mortality outcomes, but exposure-specific event counts were not fully extractable for the main pooled comparisons. Linganathan et al. [16] evaluated sarcopenia within a prediction model, but independent exposure-specific event data were unavailable for pooling. These studies therefore contributed to the narrative interpretation rather than the main event-count meta-analyses.

Strengths and limitations

This review has several strengths. Its core evidence base focused specifically on emergency abdominal surgery, including emergency laparotomy, rather than combining these patients with broad elective surgical populations. It distinguished muscle quantity from muscle quality, improving the clinical and methodological interpretation of different CT-derived measurements. It also differentiated emergency laparotomy-specific evidence from broader emergency abdominal surgery and supplementary laparotomy evidence. Finally, combining event-based meta-analysis with narrative synthesis allowed clinically relevant studies without fully extractable event counts to contribute to the overall interpretation without being inappropriately incorporated into pooled estimates.

Several limitations should be acknowledged. Most included studies were observational and retrospective, limiting causal inference. The review was not prospectively registered because screening had already commenced when registration was considered; consequently, prospective external verification of prespecified methods was not available. The review was also restricted to English-language full-text reports, and a formal GRADE assessment was not performed. Certainty of evidence was therefore interpreted narratively based on study design, consistency of findings, risk of bias, sample size, directness, clinical applicability, and methodological heterogeneity.

CT measurement approaches and diagnostic thresholds varied substantially and may have contributed to heterogeneity in pooled estimates. Some studies used psoas-derived measurements, whereas others evaluated total L3 skeletal muscle area, muscle attenuation, or composite measures [13-22]. Terminology also varied: several studies used sarcopenia to denote CT-defined low muscle quantity without a muscle-strength criterion, which differs from contemporary consensus diagnostic frameworks. The review therefore retained study terminology for reporting while interpreting these classifications primarily as muscle-quantity exposures.

Not all studies provided extractable event counts for every outcome. This particularly limited mortality pooling for studies reporting adjusted, model-based, or non-exposure-specific estimates and restricted quantitative synthesis of several secondary outcomes, including ICU use, hospital length of stay, discharge destination, and readmission. Such findings were summarized narratively rather than incorporated into pooled event-count analyses when the required data were unavailable. The pooled meta-analytic estimates were crude event-based odds ratios; adjusted estimates were summarized separately and could not be pooled consistently because covariate sets and modelling approaches differed between studies. Several pooled analyses contained only two or three studies, so the corresponding random-effects estimates and I² statistics should be interpreted cautiously.

The surgical populations also differed. Kubo et al. [22] evaluated emergency surgery for perforation panperitonitis and contributed to quantitative synthesis only where its emergency abdominal surgery population and outcome data were clinically comparable with the relevant pooled analysis. In contrast, Meyer et al. [20] was restricted to supplementary narrative synthesis because its mixed laparotomy wound-infection cohort was not restricted to emergency surgery. Finally, publication bias could not be reliably assessed because each pooled analysis contained fewer than 10 studies.

Implications for future research

Future research should standardize CT-derived muscle assessment in emergency surgical populations. Large prospective multicentre studies are needed to validate clinically relevant, emergency-surgery-specific thresholds separately for low muscle quantity (including study-defined CT sarcopenia) and low muscle quality or myosteatosis. Future studies should also report standardized outcomes, including 30-day mortality, 90-day mortality, 1-year mortality, Clavien-Dindo complications, ICU admission, hospital length of stay, discharge destination, and readmission.

Further research should evaluate whether automated CT segmentation can facilitate timely muscle assessment in emergency surgical practice. Studies should also determine whether adding CT-derived muscle measurements provides clinically meaningful incremental information beyond existing risk models and whether targeted perioperative pathways based on these measurements improve patient outcomes.

## Conclusions

Study-defined low muscle quantity (often termed CT sarcopenia) and myosteatosis were associated with increased mortality and severe postoperative complications after emergency abdominal surgery, including emergency laparotomy. These findings suggest that opportunistic CT-derived muscle assessment may support perioperative risk stratification in this high-risk population when used alongside established perioperative risk tools rather than as a replacement for them.

Interpretation should remain cautious because of heterogeneity in CT measurement methods, diagnostic thresholds, surgical populations, and outcome reporting. Future prospective studies should standardize CT definitions, validate emergency-surgery-specific thresholds separately for low muscle quantity (including study-defined CT sarcopenia) and low muscle quality or myosteatosis, and evaluate whether incorporating these measures into existing risk models improves clinical assessment and patient care.
